# Evaluation of the Fluorescence Polarization Assay for the Diagnosis of Brucellosis in Goat Milk

**DOI:** 10.3390/vetsci9060303

**Published:** 2022-06-20

**Authors:** Dianelys Sotolongo-Rodríguez, Ricardo Gomez-Flores, Magda Celina Navarro-Soto, Beatriz Arellano-Reynoso, Patricia Tamez-Guerra, Carlos Ramírez-Pfeiffer

**Affiliations:** 1 Departamento de Microbiología e Inmunología, Facultad de Ciencias Biológicas, Universidad Autónoma de Nuevo León, San Nicolás de los Garza C.P. 66450, Nuevo León, Mexico; dianelys.sotolongordrg@uanl.edu.mx (D.S.-R.); patricia.tamezgr@uanl.edu.mx (P.T.-G.); 2 Departamento de Inmunología, Facultad de Medicina Veterinaria y Zootecnia, Universidad Autónoma de Nuevo León, Escobedo C.P. 66054, Nuevo León, Mexico; magda.navarrost@uanl.edu.mx; 3 Departamento de Microbiología e Inmunología, Facultad de Medicina Veterinaria y Zootecnia, Universidad Nacional Autónoma de México, Circuito Exterior S/N, Ciudad Universitaria, Coyoacán C.P. 04510, Ciudad de México, Mexico; barellanoreynoso@fmvz.unam.mx; 4 Coordinación de Investigación Institucional, Universidad México Americana del Norte, Ciudad Reynosa C.P. 88640, Tamaulipas, Mexico

**Keywords:** brucellosis, fluorescence polarization assay, goat milk, diagnosis, epidemiology

## Abstract

The milk ring test is a detection assay for antibodies against *Brucella* in bovine milk. It has good sensitivity but tends to give false positive results. In this study, we standardized the application of the fluorescence polarization assay (FPA) for the detection of antibodies against *B.* *melitensis* in goat milk. We obtained negative serum and milk samples from healthy goat flocks in the northern zone of Nuevo León. Positive milk and negative, weak, and strong controls were obtained by mixing volumes of positive control serum with negative control milk. Milk samples were treated with citric acid, after which an FPA was performed. Results were then compared with the Rose Bengal test and the FPA in serum. Milk treatment allowed the quantification of antibodies in samples. Significant differences were found between the 2%, 4%, and 6% groups, compared with the control group (F3, 67 = 17.45, *p* < 0.0001) but not between the 2% and 4% groups (*p* = 0.0718). The cut-off value was 74.1 mP, with a sensitivity (Se) of 95% and a specificity (Sp) of 100%. Se and Sp values in field milk samples were 84% and 74.55%, respectively. Despite the FPA test on milk samples showed lower Se and Sp than the FPA test on serum samples, its cutoff may be adjusted. It may be recommended as a screening test in goat milk and become useful for the control and eradication of the disease.

## 1. Introduction

Brucellosis is a re-emerging zoonotic disease caused by several species of the genus *Brucella*, mainly affecting cattle, goats, sheep, and pigs. Small ruminants, particularly goats, are especially infected by *Brucella melitensis*, causing significant economic losses in animal production due to the slaughter of infected cattle and decreased meat and milk production [1,2]. This species is also considered the leading cause of brucellosis in humans [3,4].

Globally, the production of goat’s milk has increased at a faster rate than the human population (1.8% and 1.4%, respectively) and that of cow’s milk (1.8% vs. 0.2%, respectively). Goat milk consumption represents 2% of the milk produced and is particularly essential in Africa, Asia, and the Mediterranean region. In Latin America, Mexico leads the production of goat meat and milk [5], involving around 8.7 million goats distributed among 494,000 goat production units [6]. Goat milk is mainly produced in small-scale farms that complement the main agricultural and livestock activities [7]. Despite the increase in the production and goat farming, caprine brucellosis is considered a neglected disease that has become endemic [8,9].

The main diagnostic tests in goats approved by the World Organization for Animal Health are applied in serum samples [10], and the most widely used are the Rose Bengal (RBT) and the complement fixation (CFT) tests [11]. However, they have reduced specificity due to the partially purified lipopolysaccharide antigen (LPS) and they do not discriminate between antibodies derived from vaccination and those produced by natural infection [12,13]. The milk ring test (MRT) is extensively used for surveillance of bovine brucellosis in dairy cattle and is highly sensitive and inexpensive. However, it is prone to giving false positives, especially if samples are in poor condition due to mastitis and colostrum or are obtained at the end of the lactation cycle [14]. MRT has also been proven to be unreliable for anti-*Brucella* antibodies detection in goat milk, especially in pooled milk samples because it is unable to detect low levels of antibodies efficiently [15]. Furthermore, the small fat globules of goat and sheep milk cream absorb agglutinated stained *Brucella* in positive milk samples less efficiently and do not rise to form the typical ring at the top [15].

In addition, some goat milk characteristics may cause a decrease in the detection of antibodies [12]. Goats milk typically provides a higher proportion of total solids and protein, fat, and minerals than cow’s milk, [16]. The amount of proteins in goats milk is relatively lower than that of bovine milk but its proportion of serum proteins is higher [17]. α S1-casein proteins present a high polymorphism and the proportion of fatty acids are significantly affected by the species. In goats milk, the fat levels of C6: 0, C8: 0, C10: 0, C12: 0, and C18: 2 are higher than those in cows milk [18]. Previous reports have shown that the use of citric acid precipitates casein and vigorous shaking of the samples together with centrifugation precipitates fatty acids, preventing them from subsequently engulfing the conjugate, which facilitates its detection [12].

Fluorescence polarization (FP) was first described by Francis Perrin in 1926 [19], showing that the emission from a small fluorescent molecule excited by plane-polarized light is depolarized due to rotational diffusion during the lifetime of the fluorescence. Therefore, FP is used to follow biological processes that involve changes in molecular weight [20]. In 1952, Weber extended Perrin’s studies and applied FP to the study of proteins [21]. The application of FP to study antigen-antibody interactions was first developed by Dandliker and Feigen in the early 1960s [22], in which ovalbumin was labeled with fluorescein, which was used to produce antibodies. In regard to the diagnosis of brucellosis, the FPA was developed to increase sensitivity (Se) and specificity (Sp) and solve cross-reactivity problems of the conventional ELISA. This led to the development of the FPA test for *Brucella* detection that is performed in a short time using serum, whole blood, or milk from individual animals or from bulk milk tanks [23].

The FPA for the serological diagnosis of brucellosis uses OPS prepared from *B. abortus* S1119.3, hydrolyzed to an average molecular weight of 22 kD, and conjugated with FITC. It has been developed and validated for serological diagnosis of cattle, sheep, goats, bison, and cervids infected with smooth species of *Brucella* with Se and Sp close to 100% [24,25]. In addition, in milk samples the FPA has been developed for the detection of bovine milk antibodies to *B. abortus* with a Se (based on samples from positive-culture cattle) and Sp (based on cattle with no evidence of brucellosis) of 100 and 99.1% [12] respectively, and has been recommended as a milk test. However, it has not been standardized and applied for disease diagnosis in goat milk samples.

The use of milk samples instead of serum samples for the diagnosis of brucellosis may facilitate brucellosis eradication campaigns. Samples are easy to obtain, do not involve heavy manipulation, and reduce stress associated with a decreased milk productivity after manipulation of the animal. *Brucella* FPA milk tests may result in good epidemiological tools to achieve control and help in the control of the disease with an improved performance over current serological tests in milk samples. Taken together, the aim of the present study was to develop an FPA method for the detection of antibodies against *B. melitensis* in goat milk samples.

## 2. Materials and Methods

### 2.1. Schematic Overview of the Experimental Program

**Figure 1:**
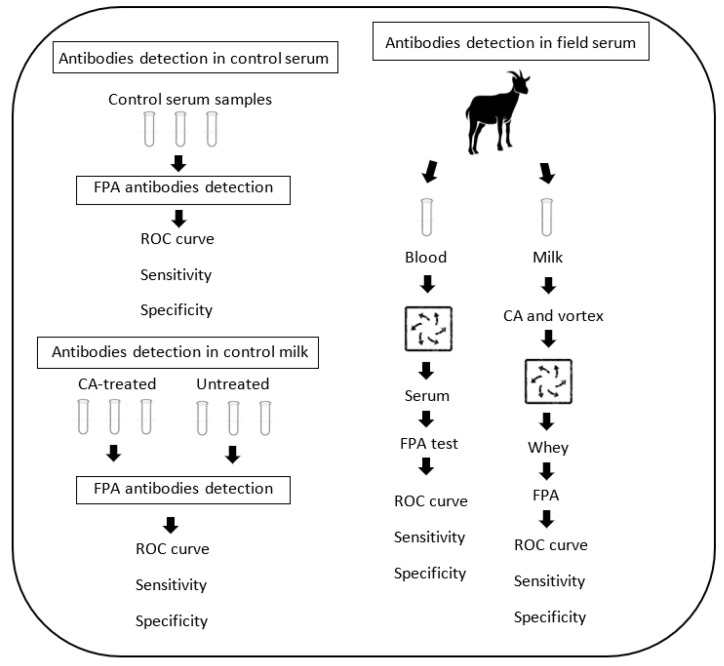
Schematic overview of the experimental program.

We obtained positive and negative control serum samples in the first stage of the experimental program (Figure 1). Next, we performed the RBT and the FPA to obtain the ROC curve and Se and Sp values. In the second stage, the FPA test was developed on treated and untreated control milk samples to detect antibodies in this matrix and whether it is dependent on milk treatment. We then obtained the ROC curve and Se and Sp values. In the third stage, we collected serum and milk field samples and performed the RBT and FPA on serum samples and the FPA on milk samples. Using the data, we obtained the ROC curve, Se, and Sp of the FPA for field milk samples.

### 2.2. Serum and Milk Controls

The Laboratorio Central Regional del Norte (LACERN), Nuevo León, México and the Facultad de Veterinaria at Universidad Nacional Autónoma de México provided us 147 positive serum samples, which were previously tested with RBT and CFT. Negative goat milk and serum samples were obtained from healthy non-vaccinated herds with official brucellosis-free accreditation in the northern areas of the State of Nuevo León, México and supplied by LACERN.

For obtaining low, weak, and strong positive milk FPA controls, different concentrations of positive control serum were mixed with two milliliters of negative goat milk. We then selected 40 µL, 80 µL, and 120 µL of serum volumes, which are the minimum amounts for antibodies detection are detected concerning the negative control, without introducing significant changes in milk characteristics.

### 2.3. Serum and Milk Field Samples

We obtained 78 blood and milk samples from goats in Tanhuato (Michoacán, México) farms, an endemic brucellosis area with no history of vaccination, which had no clinical signs of brucellosis. Samples were transported in an icebox to the Instituto de Investigaciones Agropecuarias y Forestales at Universidad Michoacana de San Nicolás de Hidalgo, Michoacán, México. Serum was separated by centrifugation at 5000 rpm/5 min and transferred to 1.5 mL vials. Serum and milk samples were stored at −20 °C and sent to the Laboratorio de Inmunología y Virología in Facultad de Ciencias Biológicas at Universidad Autónoma de Nuevo León, México.

### 2.4. Milk Treatment

Milk samples were treated as previously reported [12], with modifications. In brief, 10 µL of 1 g/mL citric acid were added to two milliliters of milk and homogenized, after which samples were mixed by vortexing for three minutes. Next, 1.8 mL of treated milk were transferred to a microcentrifuge tube and centrifuged at 5000 rpm for 10 min.

### 2.5. Serological Tests

Serum samples were tested with the FPA and the RBT, using antigen at a 3% concentration, according to the Mexican Official Norm.

### 2.6. Fluorescence Polarization Assay

We performed the FPA in 171 serum (93 control and 78 field samples) and 119 milk (41 control and 78 field samples) samples. We used a commercial kit and an FP reader, following manufacturer’s instructions. For serum samples, 20 μL of each sample and control were added to 10 mm × 75 mm borosilicate tubes containing one milliliter of FPA buffer and incubated for 15 min to 30 min at room temperature, after which basal fluorescence was measured. Next, 10 μL of FITC-labeled antigen were added to the tubes and incubated for 5 min at room temperature to obtain millipolarization (mP) values of samples and controls. We followed this procedure for milk samples, using 40 μL of untreated or citric acid-treated samples as explained above.

### 2.7. Statistical Analysis

We used the MedCalc software to determine the performance and the cut-off value of the test. The area under the curve (AUC), which is a graphical representation of the sensitivity (Se) and specificity (Sp) of a binary system, according to the variation of discrimination threshold was obtained, using the Receiver Operational Characteristics (ROC) analysis. In addition, a one-way ANOVA was used to compare means between groups.

## 3. Results

### 3.1. Fluorescence Polarization Assay on Treated and Untreated Control Milk Samples

**Figure 2:**
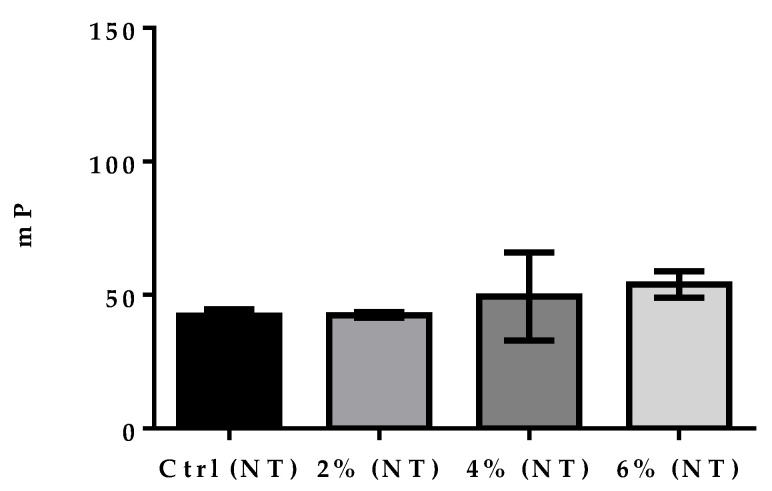
FPA on untreated control (NT) and milk samples. Data show means ± SEM of triplicate determinations from three independent experiments. Ctrl (NT), negative milk samples; 2% (NT), 4% (NT), and 6% (NT), positive milk samples. A one-way ANOVA was used to observe the difference in mP values between groups.

FPA performed on untreated milk samples did not detect significant differences between the negative milk samples group (Ctrl) and 2%, 4%, and 6% positive milk samples (F = 1.2639, *p* = 0.3504) (Figure 2).

**Figure 3:**
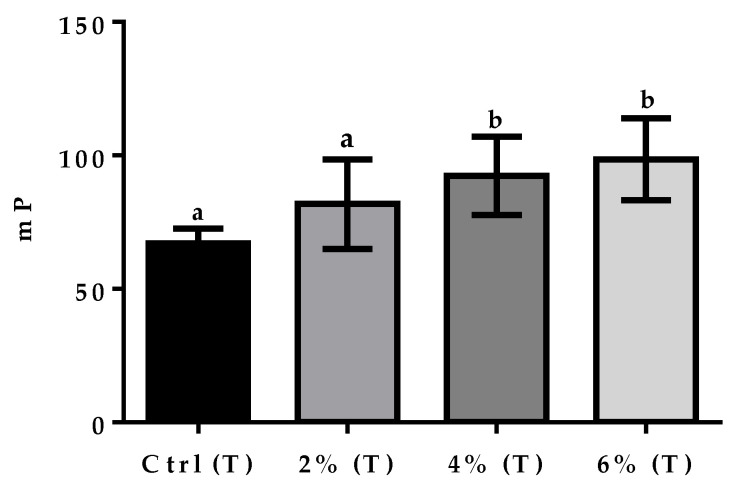
FPA on control milk samples and treatments (T). Data show means ± SEM of triplicate determinations from three independent experiments. Ctrl (NT), negative milk samples; 2% (T), 4% (T), and 6% (T), treated positive milk samples. A one-way ANOVA was used to observe the difference in mP values between groups. Bars with different letters are significantly different.

When performing the citric acid treatment described above, FPA effectively quantified antibodies in positive milk samples. Significant (F = 17.45, *p* = 0.001) differences were only observed between the 4% and 6% groups and the Ctrl (T) group (Figure 3).

### 3.2. The FPA in Control Milk Samples

**Figure 4:**
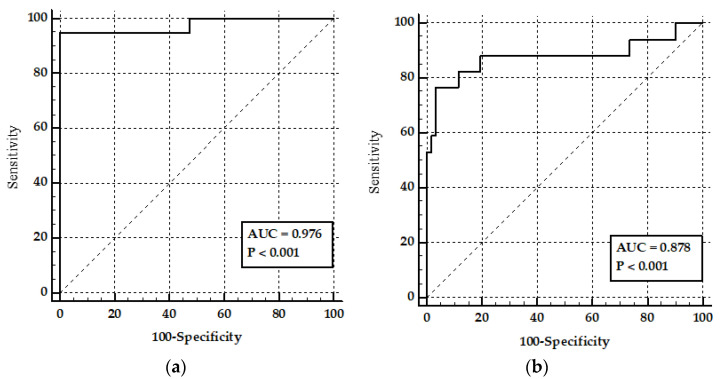
(**a**) ROC curve for the FPA test in goat milk control samples (**b**) ROC curve for the FPA test in goat milk samples obtained from areas with a high incidence of brucellosis.

**Figure 5:**
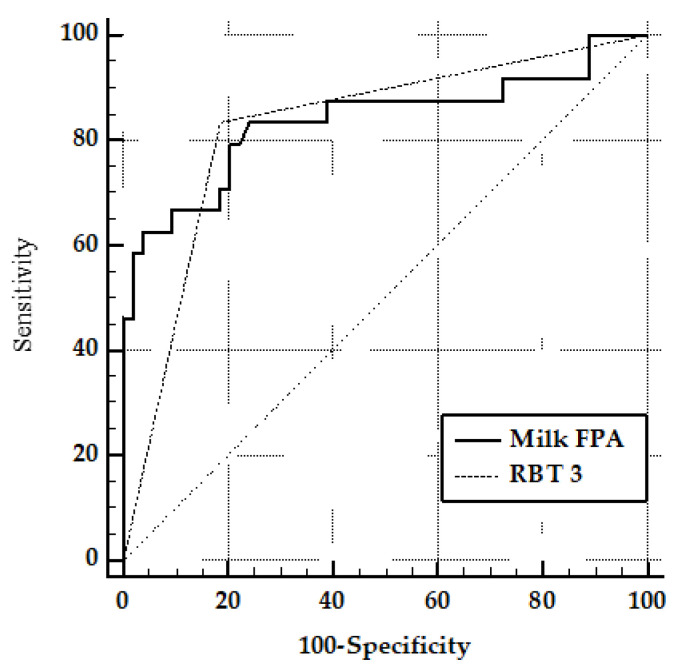
ROC of FPA performed on milk samples and the RBT 3 comparison.

We used 41 samples to determine the FPA Se, Sp, and cutoff, using an ROC analysis, based on negative (21 observations) and positive (20 observations) milk control samples (Figure 4 and Figure 5). Se and Sp with 95% confidence limits (CL) of the FPA (cutoff = 74.1 mP) were 95% (95% CL; 75.1 to 99.9 mP) and 100% (95% CL; 83.9 to 100 mP), respectively (Figure 4a).

### 3.3. The FPA on Milk Samples

When performing the FPA for 78 field milk samples, we observed a decreased Se and Sp (84% and 74.55%, respectively; 63.9% CL, 95.5 mP and 61% CL, 85.3 mP, respectively) for a cutoff value of 59.7 mP (Figure 4b).

### 3.4. Comparison of FPA and PT3 Test Performance on Milk Samples, Using the Serum FPA as the Reference Test

ROC curves analysis showed no significant differences between the AUC of the FPA and the RBT3 (Figure 5). Table 1 shows the result of the paired comparison of ROC curves. The difference between the AUC of the two tests was 1.35%, and the SEM was 0.07, with a significance level of 0.86.

### 3.5. Comparison of FPA in Serum Performance with FPA in Milk and RBT

Table 2 shows the Se, Sp, and AUC of the RBT 3, the FPA in serum samples, and the FPA in milk samples. The FPA on milk samples showed the lowest Se (84%) and the lowest Sp, compared with the FPA test on serum samples (Se: 88% and Sp: 100%) and the RBT 3 (Se: 99% and Sp: 83%).

## 4. Discussion

Brucellosis is a worldwide zoonosis of significant economic impact due to its incidence in cattle and goats [26]. Effective diagnosis and vaccination are fundamental tools for the control and eventual elimination of the disease [1]. In countries with a high incidence of brucellosis and where eradication programs are implemented, diagnosis in animals is generally achieved, using serological tests by detecting *Brucella* antibodies. Although serological diagnostic tests are available for brucellosis, most of them have reduced specificity due to the partially purified lipopolysaccharide antigen [27]. Such procedures include primary binding, precipitation, agglutination, and complement fixation tests, which are primarily applied to serum and are easier and safer to implement than the direct culture of bacteria [28], making them more feasible routine diagnostic techniques in low-income countries. The performance of serological tests varies but we do not have tests suitable for all animal species and all epidemiological settings. The diagnostic performance depends on the use of a screening test plus a confirmatory test. However, diagnosis is mainly related to antibody detection in serum [29].

The high specificity of the FPA in serum and bovine milk samples is widely reported and is attributed to the potential of eliminating the interference of vaccine antibodies and cross-reactions associated with other Gram-negative bacteria [12,13,25].

FPA is a validated test that uses the O chain of the polysaccharide of *B. abortus* conjugated to FITC as a tracer for the detection of brucellosis in serum. It is based on the random rotation of molecules in a solution, which results in the depolarization of plane polarized light [22]. This movement is inversely proportional to the molecular weight of the antigens bound to fluorescein that react with the antibodies and have a reduced movement, with the consequent reduction of the depolarization of the light. This change in the rate of depolarization is measured by the FPA in units of millipolarization (mP), providing a rapid and objective test result [22,30].

In the present study, we focused on FPA as a tool for the diagnosis of brucellosis in goat milk. Control milk samples were pre-treated with citric acid and centrifuged, which allowed to clear goat milk and the significant detection of antibodies between the control group and the 4% and 6% groups (Figure 2). We also performed FPA in positive and negative control milk samples without treatment, using the same parameters of the technique on serum samples [31]. Results obtained in these tests showed that FPA did not significantly detect *Brucella* antibodies in the groups (Figure 3). Although Nielsen et al. [32] standardized the FPA test in bovine milk without previous treatment, they recommended centrifugation of the samples to precipitate excess lipids, if a low sensitivity was observed. The presence of citric acid favors the precipitation of casein and the vigorous shaking fallowed by the centrifugation of the samples prevents the fatty acids from engulfing the conjugate, which facilitates its detection [12].

ROC analysis of the FPA performed on control milk samples showed high sensitivity (95%) and specificity (100%) with a cutoff value of 74.1 mP (Figure 4a), which positively compares with the FPA previously performed on goat serum samples. However, such results are only observed in spiked milk with antibodies, which does not always occur in a natural infection. Previous studies have shown that even in active infections using a high initial inoculum, goats may not display a significant number of antibodies throughout the infection cycle [33,34]. Although the cutoff value obtained was lower than that reported by Nielsen et al. (87.2 mP) [22] and Gall et al. (87.2 mP) [12], standardization does not imply a diagnostic cutoff [29,35]. Once the FPA was standardized in control milk samples, a second study was performed to determine the diagnostic performance of the test in goat milk, obtained from areas with a high incidence of brucellosis and no vaccination history. The heard tested had no clinical signs of brucellosis but the area had a presence of the disease in the past. FPA was used as a reference test in serum samples combined with the RBT to evaluate the diagnostic performance of FPA in milk.

The ROC analysis of the FPA in field milk samples showed an AUC of 0.878, Se of 76.4%, and a Sp of 96.7% with a cutoff value of 64.1 mP (Figure 4b). The performance of the FPA in goat milk samples has been poorly reported and is only comparable with serological studies done in cattle. The presumptive diagnosis of brucellosis, using the MRT, is a diagnostic test prescribed by the OIE. However, the MRT is non-specific when analyzing the milk of animals with mastitis or animals at either end of the milking cycle. Nicoletti et al. in 1969 showed that the MRT correctly identified 88.5% of the animals in which *B. abortus* was isolated and 77.4% of the animals in which *B. abortus* was not isolated [36]. However, lower sensitivity and specificity (72% and 80%) have been reported in bovine milk samples [37]. MRT is less sensitive for the detection of *B. melitensis* infections due to the difference between the physiological properties of goat and cow’s milk [14].

The FPA Se in field goat milk samples was similar to that reported by Gall et al. (76.9%; [12]) and Nielsen et al. (82.2%; [30]) in bovine milk. Several studies have reported that Se of FPA is lower than that of competitive ELISA (cELISA) and RBT, respectively [24,38,39]. Low concentration of antibodies in goat milk also affects the test Se [14,35]. However, the FPA maintains favorable values of Sp in comparison with conventional tests such as RBT, BPAT, and indirect ELISA (iELISA) [40,41].

In recent years, some studies have been focusing on new approaches searching for epitopes to diagnose infectious diseases such as immunoinformatic-based prediction or scanning of major antigen proteins [42]. In the case of brucellosis, increased interest has been shown in the use of the outer membrane proteins omp10, omp16, omp19, omp25, and omp31 to improve diagnosis performance by serological methods [43,44] but the detection of positive serum by protein alone is still limited [45]. The use of recombinant proteins such as BP26 has evidenced promising results with a diagnosis accuracy of 96.45% in humans and 95% in goats but has shown cross-reactivity with *Vibrio parahaemolyticus*, *Listeria monocytogenes*, *Legionella pneumophila*, *Salmonella*, and *Vibrio parahaemolyticus* [45]. However, such studies are yet to be demonstrated or standardized in milk samples.

Comparison between ROC curves obtained in the FPA test and the RBT in field milk and serum samples (Figure 5) showed no significant differences (Table 1), which may indicate no differences in the use of the FPA test in milk samples and the RBT in serum. Similar results have been observed in pig serum samples, where Se and Sp values between RBT and FPA (Se: 94.83, Sp: 99.27 and Se: 96.06, Sp: 99.54, respectively) were comparable [46]. FPA in goat milk showed lower Se and Sp than FPA and RBT3 in serum or bovine milk (Table 2). However, its cutoff may be adjusted and the results may be used as a screening test or as a surveillance tool in geographic areas where blood sampling is difficult and sampling is restricted to bulk tanks. It may also be used where milk from different flocks is collected from an area before being sent to industrial processes. Regarding the centrifugation process, it may add some time to the technique but it is still less time-consuming and requires fewer reagents than conventional tests (RBT, CFT, and ELISA) in serum samples, maintaining similar Se and Sp values than the ELISA in milk.

## 5. Conclusions

In this study, we used the FPA in goat milk samples for *Brucella* detection. Our results suggest that the test procedure may be recommended as an epidemiological brucellosis test, because it has the advantage of being standardized on a large scale and used as a test with a minimum of milk treatment, whose results may improve the diagnosis of brucellosis and contribute to the control and eradication of this disease.

## Figures and Tables

**Table 1:** Comparison of the AUC of FPA in milk and RBT.

Milk FPA versus RBT 3	
Difference between areas	0.0135
SEM	0.0766
95% Confidence interval	−0.137 to 0.164
z Statistic	0.176
Significance level	*p* = 0.8601

**Table 2:** Sensitivity and specificity of PT3, FPA in serum, and FPA in milk.

TEST	SE	95% CI	SP	95% CI	AUC
RBT 3	99.26	95.9–100.0	83.33	51.6–97.9	0.913
SERUM FPA	88.89	73.9–96.9	100.0	93.6–100.0	99.26
MILK FPA	83.33	62.6–95.3	75.93	62.4–86.5	0.878

## Data Availability

The data presented in this study are available on request from the corresponding author. The data are not publicly available due to intellectual property and confidentiality issues from our University and the Laboratorio Central Regional del Norte, Guadalupe, Nuevo León, México that facilitated milk and serum samples to our study.
